# Comparison of data mining algorithms and regression method for prediction of live body weight using biometric traits of non-descript indigenous chickens

**DOI:** 10.1007/s11250-026-05391-1

**Published:** 2026-10-18

**Authors:** Joas Albino Tsenane, Thabang Sako, Madumetja Cyril Mathapo, Thobela Louis Tyasi, Obert Tada

**Affiliations:** Department of Agricultural Economics and Animal Production, University of Limpopo, Private Bag X1106, Sovenga, Limpopo, 0727 South Africa

**Keywords:** CHAID, Shank circumference, Regression, Weighing scale, Chickens

## Abstract

The study aimed to investigate the relationship between live body weight and biometric traits, and to estimate live body weight from biometric traits of non-descript indigenous chickens. A total of 400 non-descript indigenous chickens (113 males and 287 females) were randomly selected from a multi-stage purposive sampling procedure following a cross-sectional study design. Live body weight (LBW), body length (BL), beak length (BKL), shank length (SL), shank circumference (SC), and comb height (CH) were measured. Data was analysed using Pearson’s correlation, CHAID, CART, and stepwise linear regression. Correlation results revealed that LBW of males had a positive and high correlation (*P* < 0.01) with BL (*r* = 0.63), and LBW of females had a significant positive correlation (*P* < 0.01) with SC (*r* = 0.45). Regression results showed that for males, model combining BL, BKL, CH and SC (R^2^ = 0.62, RMSE = 15.26, AIC = 2183.27, BIC = 2199.23) was best in estimation of LBW, while for females, model combining SC, BL, BKL and CH (R^2^ = 0.36, RMSE = 12.31, AIC = 2011.20, BIC = 2027.17) was best in estimation of LBW. CHAID results revealed that SC was the best variable to estimate LBW, while CART model revealed that sex was the best variable for estimation of LBW. CHAID model (*r* = 0.79, R^2^ = 0.63, RMSE = 11.73, AIC = 1969.45, BIC = 1973.44) outperformed stepwise regression and CART models. The study concludes that there was no model that had a good performance to predict live body weight of the chickens.

## Introduction

Chicken production is one of the most popular poultry husbandry systems worldwide (Habashy et al. 2021; Bila 2025). Non-descript indigenous chickens are renowned for their exceptional adaptation to harsh environmental circumstances (Hirwa et al. 2019). Indigenous chickens are a great source of protein, minerals, and vital vitamins and in addition, they contribute significantly to the household income at a rural level (Urooj et al. 2023). For breeding purposes, chicken breeders identify biometric traits that account for live body weight variations to improve growth patterns of chickens (Tyasi et al. 2021). Biometric traits are often used to characterize, explain growth, and conservation of indigenous chickens (Isaac and Ernest 2025; Bila 2025). Weighing the live body weight of animals is the most effective for famers and breeders to make good decisions for management, health, production, and marketing and it directly correlates with the productivity and profitability of chicken business (Assan 2013; Mathapo et al. 2024). However, most subsistence farmers face a challenge due to lack of weighing scales which makes it difficult for them to evaluate the growth of their chickens and to make good management choices such as administering of medicine, selective breeding, and feeding (Negash 2021; Matsambu et al. 2025). Statistical methods such as regression and data mining algorithms can be employed to estimate live body weight from biometric traits to evaluate growth patterns of animals (Kebede et al. 2021; Mathapo et al. 2022; Ebong et al. 2023). Stepwise regression analysis has the ability to detect most influencing variable, makes it a widely used and preferred method for adopting the most suitable and best regression model (Landau and Everitt 2003; Smith 2018). Data mining algorithms are collection of computations and heuristics that use data to generate a data mining model for prediction of a dependent variable (Joseph et al. 2016; Tyasi et al. 2020a, b). There have been some studies that were conducted to estimate live body weight of chickens using biometric traits (Sonubi et al. 2017; Ikeh and Okwesili 2021; Chrysostome et al. 2024). However, there have been limited studies that was conducted to estimate live body weight from biometric traits of non-descript indigenous chickens at Polokwane Local Municipality, Limpopo province of South Africa. Thus, objectives of the current study were: (1) To investigate the relationship between biometric traits and live body weight of non-descript indigenous chickens at Polokwane Local Municipality, Limpopo province of South Africa; (2) To estimate live body weight from biometric traits of non-descript indigenous chickens using stepwise linear regression model, classification and regression tree (CART) model, and chi-squared automatic interaction detection (CHAID) model; (3) To compare the predictive performance of the stepwise regression, CART, and CHAID for establishment of the best model. The findings of this study may assist farmers and breeders in estimating and improving the live body weight of indigenous chickens, thereby supporting better management and breeding decisions.

## Materials and methods

### Study area

The study was conducted in Polokwane Local Municipality, Capricorn District, Limpopo Province, South Africa. Polokwane Local Municipality’s coordinates are 23°54′ S, 29°26′ E. The Koppen Climate Classification system classified Polokwane Local Municipality as semi-arid (Kutu and Asiwe 2010). In this area, summer maximum temperature ranges from 21 °C to 22 °C, while winter average drops to 11 °C (Moshia et al. 2008). This area has a dry climate in winter, and a wet season in the summer (Alabi et al. 2013). The villages mostly use smallholder agricultural practices with modest levels of production technology (Moshia et al. 2008). The average yearly rainfall is around 495 millilitres (Phefadu and Kutu 2016).

### Sample size and sampling procedure of selecting villages

The total number of villages that form part of Polokwane Local Municipality were 143 based on information from Polokwane Local Municipality. According to Fogelman and Comber (2002), at least 1:10 ratio (10%) of the entire number of villages must be used for survey study. Therefore, 14 villages from Polokwane Local Municipality were selected. The following is formula used to calculate sample size of selected villages:

$$\mathrm{n} = \mathrm{N}^*\mathrm{sampling}\%$$

Where:

N is population size,

n is sample size,

sampling % is 10%.

Multi-stage sampling together with purposive, and random sampling procedures were utilized in the study. Multi-stage sampling is the method of collecting samples in multiple phases, with a smaller sample size at each stage (Sedgwick 2015). Purposive sampling procedure allows judgement to be employed to select situations that allow study objectives to be tackled (Mathapo et al. 2022). Random sampling is a procedure where every unit in the sample has a predetermined probability of being selected (Singh and Masuku 2014). Based on Desta et al. (2013), settlements near towns were avoided owing to their high populations of exotic chicken breeds, as well as reducing the impact of urban-affiliated agricultural systems on the usual rural village-based conventional semi-intensive system. The study followed the procedure by Hirwa et al. (2019). Therefore, stage 1: Polokwane Local Municipality was purposively selected based on the high population of non-descript indigenous chickens from Capricorn district. Stage 2: Fourteen (14) villages from the 4 regions of Polokwane Local Municipality; Northern (Makotopong, Ga-Hlahla, Ga-Semenya, Sebayeng), Eastern (Ga-Mmamatsha, Ga-Ramphere, Ga-Molepo, Ga-Mothapo), Southern (Ga-Chuene, Ga-Maja, Ga-Thaba) and Western (Ga-Mamadia, Setotolwane, Mabokelele) were purposively selected based on information from the agricultural extension officers working with those non-descript indigenous chickens. Stage 3: From the 14 selected villages, at least 5 non-descript indigenous chicken farmers were randomly selected with a minimum of 5 chicken within each village. A total number of 80 non-descript indigenous chicken farmers were selected in the study.

### Experimental animals, management, and study design

This study was conducted using 400 matured non-descript indigenous chickens (113 males and 287 females) aged from 20 weeks to two years based on availability, following the procedure outlined by Moto and Rubanza (2023). This was challenging to confirm the exact age, since farmers do not keep records. Indigenous chicken breeds have lived in rural villages throughout Africa for centuries and have adapted to the local environmental conditions (Bettridge et al. 2018). The current study employed a cross-sectional design. A cross-sectional design allows collection of data from a population once at a certain point in time (Zangirolami-Raimundo et al. 2018). The chickens were managed under semi-intensive production system. They were allowed to go out and scavenge during the day and be closed in the cage housing in the evening. The chickens were supplemented with leftovers feed based on availability at each household with no specific diet nor nutrition.

### Data collection

Biometric traits such as live body weight (LBW), and body length (BL), beak length (BKL), shank length (SL), shank circumference (SC) and comb height (CH) using tailor measuring tape in centimetres (cm) were measured following the recommendations of Yussif et al. (2023). Live body weight (LBW) was measured using a weighing scale in grams (g) (Kern and Sohn Precision balance PFB max = 3000 g, Made in Germany). Sex of the chickens was observed visually, and age was not categorised due to farmers lack of precise age of their chickens. BL was measured from the insertion of the neck into the body to the beginning of the tail at the back side of the bird. BKL was measured from the tip of the beak to the end of the beak. SL was measured from the hock joint to the metatarsal pad. SC was taken as the distance around the shank of the bird. CH was measured from the attachment of the comb at the centre base of the head to the last spike. Physical restraint was applied to prevent movement as described by FAO (2012). All measurements were taken by the same person to avoid individual variations.

### Statistical analysis

Statistical Package for Social Sciences (IBM SPSS) version 30.0.0 software was used for data analysis. Before the analysis, the data was tested for normality, heteroscedasticity, and multicollinearity following procedures of (Mutandwa 2015; Mardiatmoko 2024). Skewness and kurtosis, histogram of regression standardized residuals, and normal probability–probability (P–P) plot of regression standardized residuals for live body weight were used to test for normality of the data. Scatterplot of regression standardized residuals versus regression standardized predicted values for live body weight was used to test for heteroscedasticity. Pearson’s correlation was used to compute correlation between live body weight and biometric traits of both males and female non-descript indigenous chickens. After determining correlation, the collinearity statistics (variance inflation factor (VIF) and tolerance) were used to test for multicollinearity between live body weight and biometric traits of chickens. Stepwise regression model, Classification and regression tree (CART) and Chi-squared automatic interaction detection (CHAID) models were used for estimation of live body weight from biometric traits of non-descript indigenous chickens. The used parent node and child node ratios on CHAID and CART algorithms was 75:25. The tests were performed at a minimum of 95% level of confidence to detect their significance.

CHAID algorithm is an analysis which is non-parametric for a dependent variable with many independent variables, and it allows researchers to categorize the independent variables as predictors for the dependent variable (Celik 2019). CHAID model iteratively divides a subset into new multi-subsets made up of comparable responses to a dependent variable to create a decision tree structure starting from the root node (Eyduran et al. 2017; Tyasi et al. 2021). CRT is frequently used when the dependent variable is continuous, which aids in value prediction, and when the dependent variable is categorical, which aids in identifying the class that a dependent variable is categorized (Tyasi et al. 2020a; Assan et al. 2024).

The following stepwise regression model was used:

$$\:Y\:=\:a\:+\:b1X1\:+\dots\:+\:bnXn$$

Where:

Y is dependent variable (live body weight),

a is intercept,

b is regression coefficients of independent variables,

X is independent variables (BL, SC, CH, SL and BKL).

The best model between CHAID, CART and stepwise regression was determined by calculating the goodness of fit test criteria outlined by Celik (2019) and Matsambu et al. (2025). The following goodness of fit criteria were calculated:

Correlation Coefficient (r)

$$\:r\:=\:\frac{cov\:(Yi\:,Yip\:\:)}{Syi\:SYip\:}$$

Coefficient of Determination (R^2^)

$$\mathrm{R}^2\:=\left[1-\:\frac{{\sum\:}_{i=1}^{n}({Y}_{i}-{\hat{{Y}}_{i})}^{2}}{{\sum\:}_{i=1}^{n}({Y}_{i}-{\overline{Y}})^{2}}\right]$$

Adjusted Coefficient of determination (Adj.R^2^)

$$\mathrm{Adj.R}^2\:=\:\left[1-\:\frac{\frac{1}{N-k-1}\:{\sum\:}_{i=1}^{N}({Y}_{i}-{\hat{{Y}}_{i})}^{2}}{\frac{1}{N-1}{\sum\:}_{i=1}^{N}({Y}_{i}-\overline{Y})^{2}}\right]$$

Root Mean Square Error (RMSE)

$$\:RMSE\:=\:\sqrt{\frac{{\sum\:}_{i=1}^{N}({Y}_{i}-{\hat{{Y}}_{i})}^{2}}{N}}$$

Akaike information criterion (AIC)

$$\:AIC=\:NLn\:\left(\frac{SSE}{N}\right)\:+\:2p$$

Bayesian information criterion (BIC)

$$\:BIC=\:NLn\:\left(\frac{SSE}{N}\right)\:+\:pLn$$

Where:

Y_i_, the actual body weight (g) of the indigenous chicken;

Ŷ_i_, the predicted body weight value of the indigenous chicken;

Ȳ, average of the actual body weight of the indigenous chicken;

k, number of significant independent variables in the model;

and n, the total number of indigenous chickens.

The residual value of each indigenous chicken is expressed as Ɛ_i_ = Y_i_ – Ŷ_i_.

## Results

### Descriptive statistics for biometric traits of chickens

Table 1 shows descriptive statistics of chickens. The skewness and kurtosis results indicated that data of LBW (0.97 **±** 0.12 and 1.10 **±** 0.24, respectively), BL (0.68 **±** 0.12 and 0.40 **±** 0.24, respectively), BKL (-.

0.07 **±** 0.12 and − 0.78 **±** 0.24, respectively), SL (0.29 **±** 0.12 and − 0.34 **±** 0.24, respectively), SC (0.35 **±** 0.12 and − 0.07 **±** 0.24, respectively), and CH (0.87 **±** 0.12 and 0.80 **±** 0.24, respectively) was approximately normal.

**Table 1:** Descriptive statistics of chickens

Biometric traits	*N*	Minimum	Maximum	Mean	Std. Deviation	Skewness ± Std. Error	Kurtosis ± Std. Error
LBW (g)	400	598.40	2976.60	1370.06	482.38	0.97 **±** 0.12	1.10 **±** 0.24
BL (cm)	400	16.00	33.00	22.73	3.37	0.68 **±** 0.12	0.40 **±** 0.24
BKL (cm)	400	2.80	5.00	3.81	0.54	-0.07 **±** 0.12	-0.78 **±** 0.24
SL (cm)	400	5.30	11.0	8.37	1.25	0.29 **±** 0.12	-0.34 **±** 0.24
SC (cm)	400	3.00	6.50	4.62	0.78	0.35 **±** 0.12	-0.07 **±** 0.24
CH (cm)	400	0.50	6.30	2.97	1.19	0.87 **±** 0.12	0.80 **±** 0.24

### Normality test

**Fig. 1:**
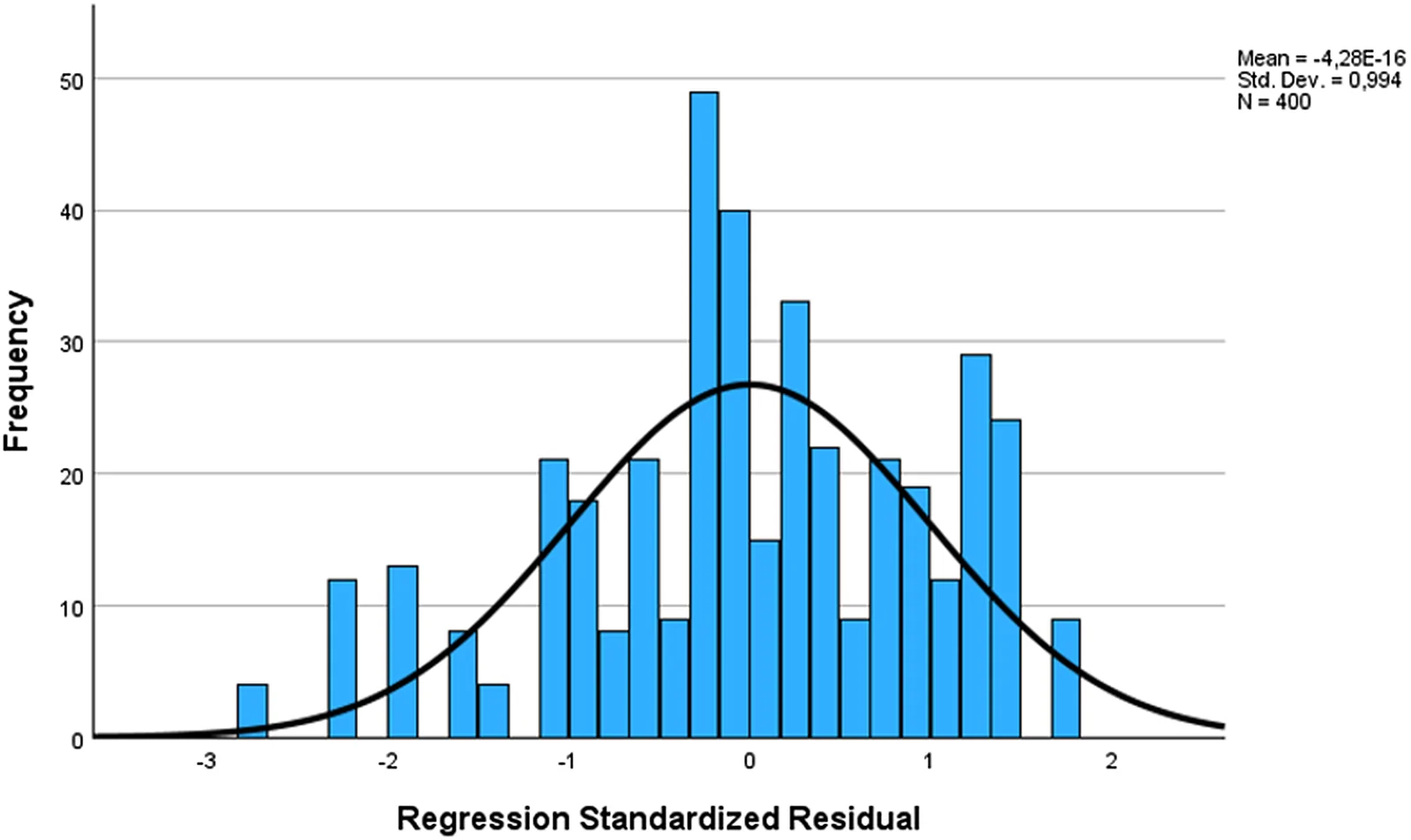
Histogram of regression standardized residuals for live body weight

Figure 1 shows histogram of regression standardized residuals for live body weight. The histogram showed an approximately normal distribution, with residuals symmetrically distributed around zero. The residual mean was close to zero (-4.28 × 10⁻¹⁶) and the standard deviation was 0.994, indicating that the assumption of normality of residuals was satisfied. No severe outliers were detected.

**Fig. 2:**
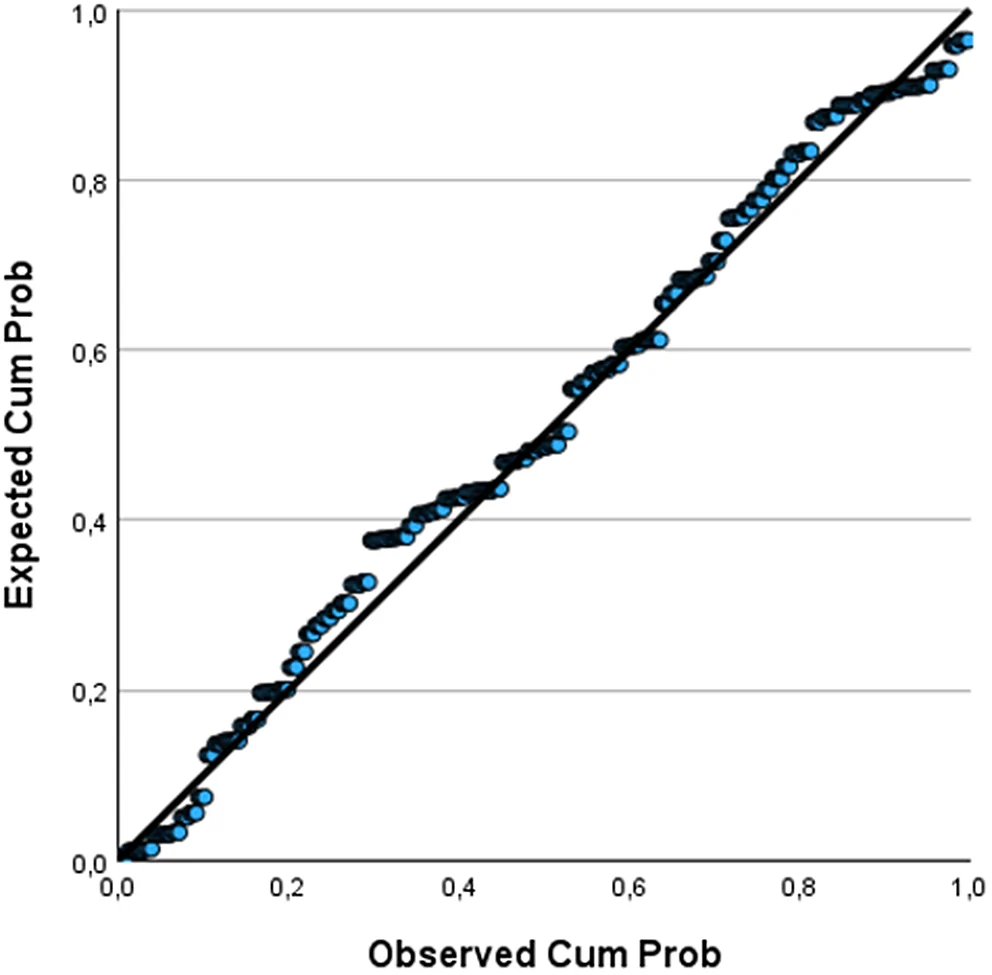
Normal probability–probability (P–P) plot of regression standardized residuals for live body weight

Figure 2 shows normal probability–probability (P–P) plot of regression standardized residuals for live body weight. The residual points closely followed the diagonal reference line, indicating that the residuals were approximately normally distributed.

### Heteroskedasticity test

**Fig. 3:**
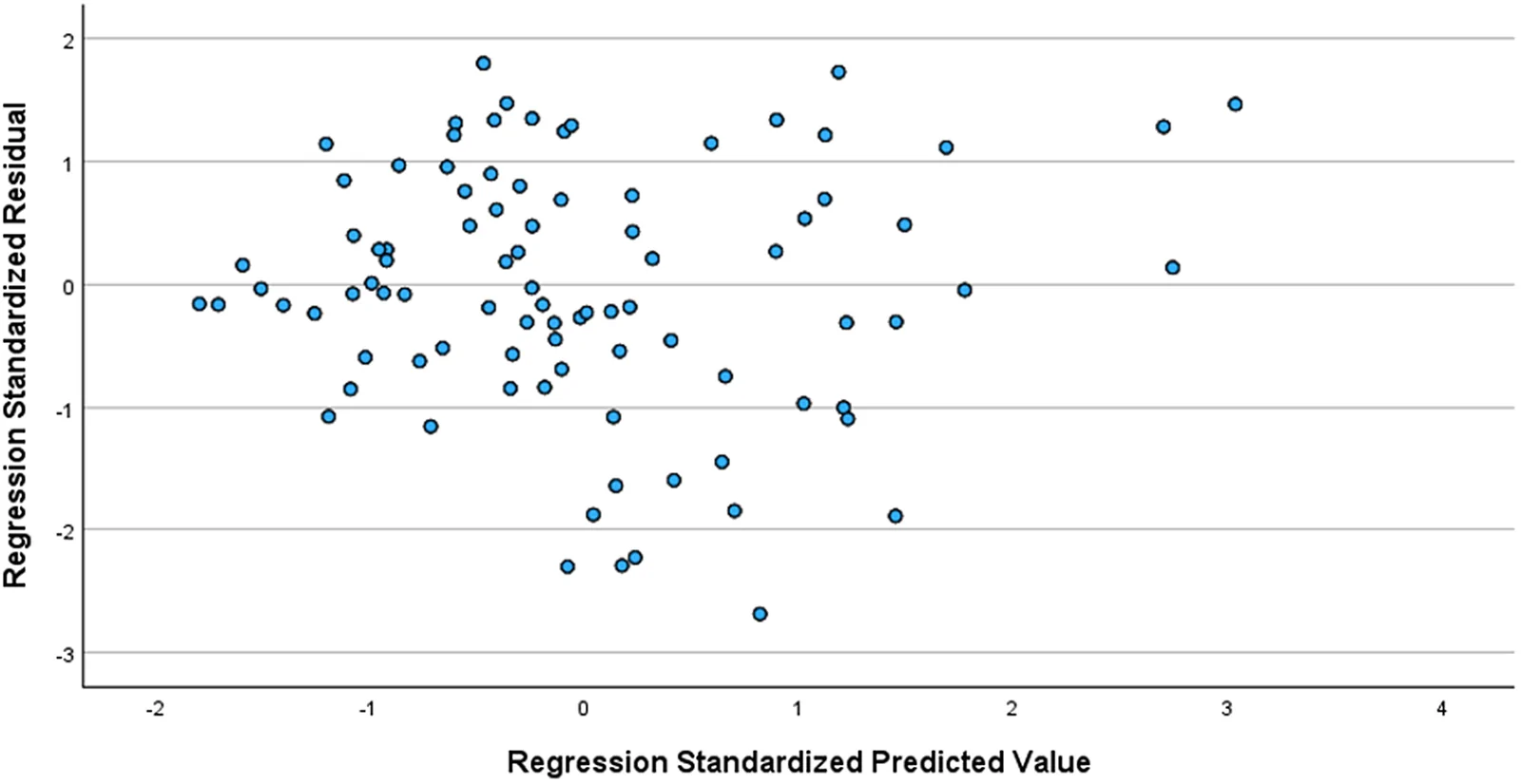
Scatterplot of regression standardized residuals versus regression standardized predicted values for live body weight

Figure 3 demonstrates a scatterplot of regression standardized residuals versus regression standardized predicted values for live body weight. The residuals were randomly distributed around zero with no evident pattern (funnel shape), indicating homoscedasticity. Few observations showed larger residuals, but no severe outliers were detected.

### Relationships between live body weight and biometric traits of chickens

Table 2 shows the relationship between live body weight (LBW) and some biometric traits. The results for males and females are displayed above and below the diagonal, respectively. The results demonstrated that LBW of males was positively and significantly correlated (*P* < 0.01) with BL, BKL, SL, SC and CH. The results indicated that LBW of females was positively and significantly correlated (*P* < 0.01) with BL, SL, SC and CH, and not significantly correlated (*P* > 0.05) with BKL.

**Table 2:** Correlation between live body weight and some biometric traits of males above the diagonal and females below the diagonal

Traits	LBW	BL	BKL	SL	SC	CH
LBW		0.63^**^	0.27^**^	0.40^**^	0.47^**^	0.55^**^
BL	0.33^**^		− 0.21^*^	0.35^**^	0.33^**^	0.49^**^
BKL	− 0.08^ns^	− 0.25^**^		0.27^**^	0.26^**^	0.06^ns^
SL	0.26^**^	0.4.6^**^	-0.04^ns^		0.04^ns^	0.40^**^
SC	0.45^**^	0.05^ns^	0.30^**^	0.13^*^		0.25^**^
CH	0.37^**^	− 0.01^ns^	0.24^**^	0.07^ns^	0.59^**^	

* = significant at P < 0.05, ** = highly significant at P < 0.01, ^ns^ = not significant at P > 0.05, LBW = live body weight, BL = body length, BKL = beak length, SL = shank length, SC = shank circumference, CH = comb height.

### Multicollinearity test

Table 3 indicates multicollinearity testing between live body weight and biometric traits. Tolerance and VIF values ranged from 0.482 to 0.723 and 1.384 to 2.073, indicating absence of multi-collinearity between LBW and biometric traits.

**Table 3:** Multicollinearity testing between live body weight and biometric traits

Biometric traits	Collinearity Statistics	
	Tolerance	VIF
Body length	0.636	1.574
Beak length	0.723	1.384
Shank length	0.644	1.553
Shank circumference	0.489	2.044
Comb height	0.482	2.073

VIF = variance inflation factor

### Live body weight estimation from biometric traits of chickens using stepwise regression

Estimation of live body weight of chickens from biometric traits using stepwise regression is shown on Table 4. The results showed that for males, coefficient of determination (R^2^) ranged from 0.39 to 0.62, with the model combining BL, BKL, CH and SC (model 4) had the highest coefficient of determination (R^2^ = 0.62), and the lowest root mean square of error (RMSE = 15.262). The results indicated that on females, coefficient of determination ranged from 0.20 to 0.36, and the model combining SC, BL, BKL and CH (model 4) had R^2^ of 0.36 with the least root mean square of error (RMSE = 12.309).

**Table 4:** Estimation of live body weight of chickens from biometric traits

Model number	Model	*R*	*R* ^2^	Adj.*R*^2^	RMSE	AIC	BIC	*P* value
Male								
1	LBW = − 139.42 + 81.98BL	0.625	0.390	0.385	18.941	2353.039	2357.031	< 0.01
2	LBW = − 2282.71 + 93.70BL + 45.82BKL	0.747	0.558	0.550	16.225	2230.233	2238.215	< 0.01
3	LBW = − 2108.11 + 77.16BL + 406.62BKL + 98.98CH	0.774	0.599	0.588	15.547	2197.083	2209.058	< 0.01
4	LBW = − 2243.76 + 69.32BL + 348.78BKL + 96.2324CH + 106.95SC	0.786	0.618	0.604	15.262	2183.265	2199.231	< 0.01
Female								
1	LBW = 113.34 + 245.37SC	0.449	0.202	0.199	13.574	2086.530	2090.521	< 0.01
2	LBW = − 647.61 + 245.67SC + 34.55BL	0.560	0.313	0.309	12.625	2029.521	2037.504	< 0.01
3	LBW = − 353.44 + 270.49SC + 30.70BL − 85.80BKL	0.577	0.333	0.326	12.483	2021.486	2033.461	< 0.01
4	LBW = − 260.41 + 213.33SC + 30.65BL – 93.43BKL + 73.492CH	0.596	0.355	0.346	12.309	2011.201	2027.167	< 0.01

LBW = live body weight, BL = body length, BKL = beak length, SL = shank length, SC = shank circumference, CH = comb height, r = correlation coefficient, R^2^ = coefficient of determination, Adj.R^2^ = adjusted coefficient of determination, RMSE = root mean square of error, AIC = Akaike information criterion, BIC = Bayesian information criterion, P value = probability value

### Estimation of live body weight from biometric traits using CHAID model

**Fig. 4:**
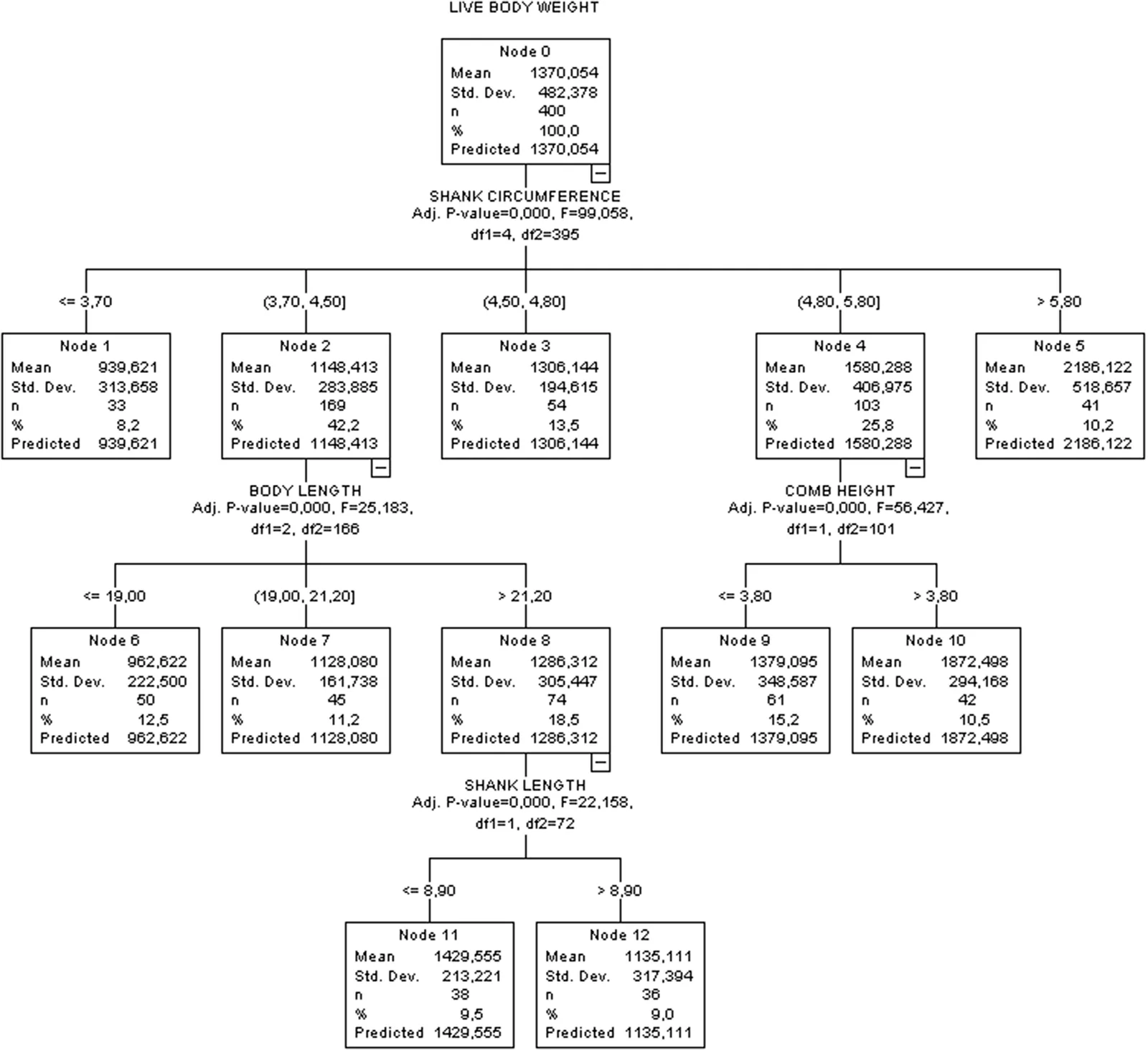
Regression decision tree diagram for prediction of body weight of chickens using the CHAID algorithm

Figure 4 shows the regression tree diagram designed by CHAID model. Node 0 displayed the summary of live body weight of chickens (1370.05 ± 482.378 g; *n* = 400). Node 0 was split based on shank circumference (SC) into five subgroups: Node 1 (SC ≤ 3.70 cm; 939.621 ± 313.658 g; *n* = 33), Node 2 (SC: 3.70–4.50 cm; 1148.413 ± 283.885 g; *n* = 169), Node 3 (SC: 4.50–4.80 cm; 1306.144 ± 194.614 g; *n* = 54), Node 4 (SC: 4.80–5.80 cm; 1580.288 ± 406.975 g; *n* = 103) and Node 5 (SC > 5.80 cm; 2186.122 ± 518.657 g; *n* = 41). Node 2 was split based on body length (BL) into three subgroups: Node 6 (BL ≤ 19.00 cm; 962.622 ± 222.500 g; *n* = 50), Node 7 (BL: 19.00–21.20 cm; 1128.080 ± 161.738 g; *n* = 45), Node 8 (BL > 21.20 cm; 1286.312 ± 305.447 g; *n* = 73). Node 5 was split based on comb height (CH) into two subgroups: Node 9 (CH ≤ 3.80 cm; 1379.095 ± 348.587 g; *n* = 61), Node 10 (CH > 3.80 cm; 1872.498 ± 294.168 g; *n* = 42). Node 8 was split based on SL into two subgroups: Node 11 (SL ≤ 8.90 cm; 1429.555 ± 213.221 g; *n* = 38) and Node 12 (SL > 8.90 cm; 1135.111 ± 317.394 g; *n* = 36).

### Estimation of live body weight from biometric traits using CART model

**Fig. 5:**
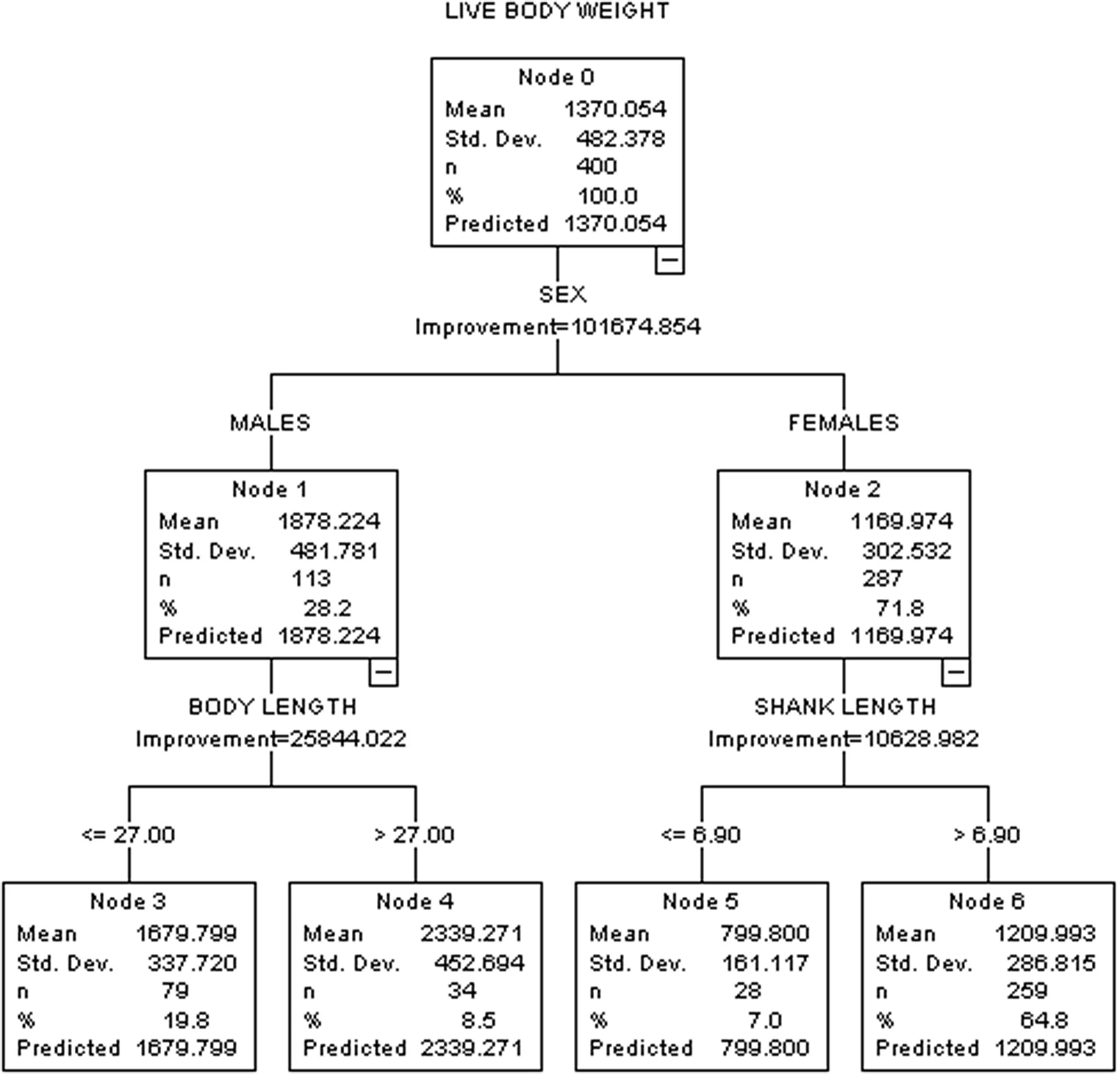
Regression decision tree diagram for prediction of body weight of chickens using the CART algorithm

Figure 5 shows a tree diagram for estimation of body weight obtained from CART model. Node 0 showed the summary of live body weight of chickens (1370.054 ± 482.378 g; *n* = 400). Node 0 was split into two subgroups based on sex of the chickens: Node 1 (males; 1878.228 ± 481.781 g; *n* = 113) and Node 2 (females; 1169.974 ± 302.532 g; *n* = 287). Node 1 was split into two subgroups based on Body length (BL): Node 3 (BL ≤ 27.00 cm; 1679.799 ± 337.720 g; *n* = 79) and Node 4 (BL > 27.00 cm; 2339.271 ± 452.694 g; *n* = 34). Node 2 was split into two subgroups based on shank length (SL): Node 5 (SL ≤ 6.90 cm; 799.800 ± 161.117 g; *n* = 28) and Node 6 (SL > 6.90 cm; 1209.993 ± 286.815 g; *n* = 259).

### Predictive performance of Stepwise regression, CHAID, and CART

Table 5 shows the goodness of fit for comparison for performance of stepwise regression, CHAID, and CART models. The results indicated that CHAID model outperformed stepwise regression and CART models with the highest correlation coefficient (*r* = 0.790) coefficient of determination (R^2^ = 0.625), and adjusted coefficient of determination (Adj.R^2^ = 0.624) and with the lowest values of root mean square of error (RMSE = 11.726), Akaike information criteria (AIC = 1969.450) and Bayesian information criterion (BIC = 1973.441).

**Table 5:** Predictive performance of the models

Models	*R*	*R* ^2^	Adj.*R*^2^	RMSE	AIC	BIC	*P* value
**Stepwise regression**							
**Male**							
Model 1	0.625	0.390	0.385	18.941	2353.039	2357.031	< 0.01
Mode 2	0.747	0.558	0.550	16.225	2230.233	2238.215	< 0.01
Mode 3	0.774	0.599	0.588	15.547	2197.083	2209.058	< 0.01
Mode 4	0.786	0.618	0.604	15.262	2183.265	2199.231	< 0.01
**Female**							
Mode 1	0.449	0.202	0.199	13.574	2086.530	2090.521	< 0.01
Mode 2	0.560	0.313	0.309	12.625	2029.521	2037.504	< 0.01
Mode 3	0.577	0.333	0.326	12.483	2021.486	2033.461	< 0.01
Mode 4	0.596	0.355	0.346	12.309	2011.201	2027.167	< 0.01
**CHAID**	0.790	0.625	0.624	11.726	1969.450	1973.441	< 0.01
**CART**	0.771	0.595	0.594	11.884	1980.116	1984.108	< 0.01
**Decision**	Greater is better	Greater is better	Greater is better	Lower is better	Lower is better	Lower is better	Significant is better

r = correlation coefficient, R^2^ = coefficient of determination, Adj.R^2^ = adjusted coefficient of determination, RMSE = root mean square of error, AIC = Akaike information criterion, BIC = Bayesian information criterion, P value = probability value

## Discussion

Live body weight of chickens is regarded as a trait of economic importance, and its estimation is vital to small scale farmers (Malomane et al. 2014). Pearson’s correlation was used to detect the relationship between the biometric traits and live body weight of chickens. The results revealed that live body weight of both sexes was positive and significantly correlated with body length, shank length, shank circumference and comb height. Body length is one of the most fundamental traits contributing to the overall body size and weight of chickens, reflecting to muscle and fat deposition (Chen et al. 2025). Shank length and circumference are important in supporting the upper body weight of chickens, and thus their growth is positively related to the overall live body weight (Ikeh and Okwesili 2021). Furthermore, live body weight on males was positive and significantly correlated with beak length, however it was not significantly correlated on females. Male chickens use their beaks to scavenge for food, fight against predation, and compete amongst themselves for mating rights (Carvalho et al. 2022). The findings of the current study are consistent with findings of Yunusa and Adeoti (2014), that detected live body weight of both male and female Fulani, and Yoruba chickens had a significant relationship with body length. The findings of Yakubu and Ari (2018) are consistent with the findings of the current study, where live body weight had a significant correlation with male shank length and circumference of Sasso, Kuroiler and Fulani chickens. Udeh et al. (2021) reported similar results with current study, where live body weight of male and female Nigerian heavy local chicken ecotype had a significant correlation with shank length and body length. The findings of the current study were in contrast with those of Ikeh and Okwesili (2021) and Adenaike et al. (2015), that reported a significant and high relationship between live body weight and shank length of indigenous chickens. The difference in the results may be due to different environmental conditions that the chickens are exposed. The association found between biometric traits (comb height and shank circumference) and body weight implies that those traits may be controlled by same gene.

Pearson’s correlation results are used to detect the relationship between biometric traits and live body weight (Tyasi et al. 2024). However, they do not focus on the prediction of live body weight (Assan et al. 2024). Thus, stepwise regression, CHAID, and CART models were used to estimate live body weight of chickens from selected biometric traits. Regression findings revealed that, for males, the models explained thirty-nine to sixty-two% of variation in live body weight. The model with combination of body length, beak length, comb height, and shank circumference showed the highest variation on the prediction of live body weight. In females, the variation explained in predicting live body weight ranged from twenty to thirty six%. However, the model that included shank circumference, body length, beak length, and comb height explained the highest variation of live body weight. According to Mathapo et al. 2022; at least 70% (70%) of coefficient of determination is adequate to validate a good performing model. Therefore, all the models showed poor performance with variation towards live body weight which is less than 70%. The findings of the current study are consistent with those reported by Nweke-Okorocha and Gunn (2022), which revealed that a model including shank circumference and body length was significant in estimation of live body weight. However, the model had higher variation in live body weight estimates as compared to the models of the current study. This implies that Fulani chickens have different biological attributes with the non-descript indigenous chickens at Polokwane Local Municipality. The differences in the results could be due to adaptation to different climatic conditions. The findings of Habashy et al. (2021) are consistent with the findings of the current study based on 62% variation in body weight estimates of Egyptian Golden Sabahia male Chickens on the model that consist of body length. The findings of the current study are in contrast with the findings of Obike et al. (2019), that reported less than 20% of variation in live body weight estimates of Noiler hybrid strain and Yoruba ecotype chickens on the model including body length at ten to sixteen weeks of age. The difference in the results could be due to the current study having used chickens of over twenty weeks of age. The results imply that although the models have weak predictive performance, the combination of body length, beak length, comb height, and shank circumference can be used to improve live body weight of chickens.

The CART and CHAID algorithms were further used to estimate live body weight of chickens in the absence of weighing scales. CHAID findings revealed that shank circumference, body length, shank length, and comb height were the traits that can be used to estimate live body weight of non-descript indigenous chickens. Furthermore, the model revealed that shank circumference can be used as a single trait for prediction of body weight of non-descript indigenous chickens. Shank circumference is considered a reference index that is valuable to measure breeding value in chickens (Zheng et al. 2022). CART findings revealed that sex, body length and shank length can be used as predictor of live body weight of non-descript indigenous chickens. The model further revealed that sex can be used as single predictor variable for live body weight. According to Benyi et al. (2015), sex is one of the most influencing parameters to the variation in live body weight of chickens. Findings of the current study were consistent with the findings of Assan et al. (2024), who reported that sex can be a variable to estimate live body weight Sacco, Astrolope, and Boschveld chickens through CART model. However, Liswaniso et al. (2025) reported inconsistent findings to the current study, which revealed that shank circumference was the best single predictor of live body weight of Kuroiler chickens using CART model. Furthermore, Isaac and Ernest (2025) and Matsambu et al. (2025), both reported inconsistent findings to current study, where CHAID model revealed that body length was the best predictor of body weight of indigenous chickens. The variances in the results could be due to differences in the breeds which influence the growth patterns of biometric traits. This implies that shank circumference and sex are strong variables for prediction of live body weight because of their ability to explain variation in body weight of non-descript indigenous chickens.

The study further checked the performance of the models. The findings revealed that the variation towards live body weight for all the models was low (less than 70%). However, CHAID was best model for prediction of live body weight of indigenous chickens compared to stepwise regression and CART models. This was based on its highest coefficient of determination, correlation coefficient, adjusted coefficient of determination, root mean square of error, Akaike information criterion, and Bayesian information criterion. Findings of the current study were consistent with the findings of Isaac and Ernest (2025), who reported that CHAID model was the best model for predicting live body weight of indigenous chickens. Furthermore, the findings of the current study are in contrast with the results of Assan et al. (2024); Tyasi et al. (2020a), who reported that CART model was best model for estimation of live body weight of Astrolope, Boschveld and indigenous Sacco, and Hy-line silver brown and Potchefstroom Koekoek chickens, respectively. Furthermore, the findings of Billa (2023) also revealed that CART model was the best in estimation of live body weight of broiler chickens. The variation in results could be due to the different chickens’ breeds, and the environmental conditions that the chickens were raised. The results imply that CHAID was best algorithm to model the best predictor of live body weight of chickens.

In conclusion regression model with the combination of body length, comb height, shank circumference, shank length, and beak length had the highest value of 62% of variation towards live body weight of male chickens. On female chickens, variation towards live body was low, with 30% on the model with the combination of comb height, body length, and shank length. CART model concluded that sex can be used as single variable for predictor of live body weight of non-descript indigenous chickens. CHAID revealed that shank circumference is the best single predictor of live body weight of non-descript indigenous chickens. All the included models performed poorly when it comes to the prediction of body weight from biometric traits. It is recommended that other machine learning models should be used to predict body weight of indigenous chicken in future. The limitations of the current study are that; (1) Age was not included as a discriminator for live body weight due to farmers lacking data for age of the chickens. (2) Nutrition of the feed fed to the chickens was unknown as farmers provided leftover feed based on availability, and most times the chickens were scavenging. (3) The predictive models were evaluated using the same dataset used for model development, without independent validation or cross-validation and this may have resulted in overestimated predictive performance.

## Data Availability

All data collected and compiled during the current investigation are available upon request to the corresponding author.
